# A comprehensive overview of gastric cancer management from a surgical point of view

**DOI:** 10.1016/j.bj.2024.100817

**Published:** 2024-11-18

**Authors:** Jun-Te Hsu, Yu-Ning Lin, Yi-Fu Chen, Hao-Wei Kou, Shan-Yu Wang, Wen-Chi Chou, Ting-Rong Wu, Ta-Sen Yeh

**Affiliations:** a Department of General Surgery, Chang Gung Memorial Hospital at Linkou, Chang Gung University College of Medicine, Taoyuan, Taiwan; b Department of Hematology-Oncology, Chang Gung Memorial Hospital at Linkou, Chang Gung University College of Medicine, Taoyuan, Taiwan

**Keywords:** Gastric cancer, Lymphadenectomy, Minimally invasive surgery, Neoadjuvant chemotherapy, Conversion surgery, Intraperitoneal chemotherapy

## Abstract

Despite advancements in medical care, surgical technologies, and the development of novel treatments over the past decade, the prognosis for patients with gastric cancer (GC) has only modestly improved. This is primarily due to the fact that the majority of patients are diagnosed at advanced stages or present with metastatic disease. Radical resection remains the cornerstone of potentially curative treatment, yet the overall 5-year survival rate remains below 35%. The management of GC varies globally, influenced by factors such as geographical disparities, patient comorbidities and performance status, surgical approaches, and available medical resources. Multidisciplinary collaboration and a multimodal treatment approach are essential for optimizing patient outcomes. Surgeons must stay updated on emerging surgical concepts and make informed decisions regarding patient selection, timing of intervention, and the adoption of appropriate surgical techniques to improve both quality of life and prognosis. This review aims to provide a surgical perspective on the management of GC across all stages, highlighting the importance of a comprehensive treatment approach. Endoscopic resection may be a viable option for early GC in patients with minimal risk of lymph node metastasis, particularly in elderly patients with high surgical risk or severe comorbidities. For advanced GC, neoadjuvant therapy followed by surgery could be a promising strategy to improve patient outcomes. Conversion surgery offers a potential survival benefit for patients who respond to treatment with tumor downstaging. The treatment of peritoneal carcinomatosis remains challenging; however, hyperthermic intraperitoneal chemotherapy combined with complete cytoreductive surgery or pressurized intraperitoneal aerosolized chemotherapy may prolong survival or improve quality of life in highly selected patients.

## 1 Introduction

Gastric cancer (GC) remains a significant global health concern, being the fourth leading cause of cancer-related deaths and the fifth most prevalent malignant tumor worldwide, according to GLOBOCAN 2020 [1]. The incidence and prevalence of GC vary geographically, with East Asia, Central Europe, and Eastern Europe showing the highest rates, while significantly lower rates are observed in Africa and North America [2]. Despite advancements in medical care, surgical technologies, and the development of novel therapies over the past decade, the prognosis for GC patients has shown only modest improvement, primarily due to late-stage diagnoses. A number of studies have shown that high-volume centers and experienced surgeons have fewer surgical complications and better 5-year survival rates compared to their counterparts [3]. The cancer-specific 5-year survival rate ranges between 30% and 35% worldwide, except in Japan and Korea, where early GC is more frequently diagnosed through screening programs [2].

The treatment of GC necessitates a multidisciplinary approach that continues to evolve. Currently, curative surgical resection remains the cornerstone of GC management, with the extent of lymphadenectomy tailored to disease severity and tumor location [4]. For early GC with no risk or very low risk (<1%) of lymph node metastasis, endoscopic resection has been adopted as a treatment option worldwide, showing outcomes equivalent to those of surgical resection [[4], [5], [6], [7], [8]]. Beyer et al. suggest that for locally advanced disease, proximal margins of more than 3 cm are recommended for tumors with an expansive growth pattern, and more than 5 cm for lesions with an infiltrative growth pattern [9]. However, controversies still exist regarding the impact of positive resection margins and the role of re-resection on survival, as highlighted in a systematic review article [3].

There are notable differences in surgical strategies for resectable localized GC between the East and the West [10]. The major difference is that in the West, neoadjuvant chemotherapy followed by surgery is preferred, while in the East, upfront surgery is more commonly practiced. Another discrepancy is that in the East, GC is often treated at high-volume centers by experienced surgeons, whereas in the West, GC is not as centralized, is diagnosed at more advanced stages, and surgeons typically perform less aggressive surgeries. Although global care of GC is becoming more standardized, the differences in surgical strategies and practices between the East and the West have lessened over time.

Recent literature has explored the impact of adding splenectomy to gastrectomy [11,12], as well as the adoption of minimally invasive surgical techniques such as laparoscopic gastrectomy (LG) and robotic-assisted gastrectomy (RG), facilitated by significant advances in surgical technologies [[13], [14], [15]]. For patients with advanced disease, neoadjuvant therapy has emerged as a valuable approach, offering benefits such as downstaging the primary tumor, eliminating occult distant metastases, and improving R0 resection rates, ultimately leading to improved progression-free survival (PFS) and/or overall survival (OS) [[16], [17], [18], [19]]. Additionally, conversion surgery has shown promise in prolonging survival [[20], [21], [22], [23]].

While surgical management is not typically the first-line option for patients with metastatic disease, it may be employed to address cancer-related complications such as obstruction, bleeding, or perforation [24,25]. Systemic chemotherapy, targeted therapy, immunotherapy, and intra-abdominal (locoregional) treatments, such as hyperthermic intraperitoneal chemotherapy (HIPEC) combined with cytoreductive surgery (CRS) or pressurized intraperitoneal aerosolized chemotherapy (PIPEC), are also integral components of the treatment for advanced GC and metastatic GC (mGC) [[26], [27], [28], [29], [30], [31], [32], [33], [34], [35], [36]].

The heterogeneity in GC management across regions stems from geographical variations, patient factors such as comorbidities and performance status, surgical approaches, and healthcare resources. Multidisciplinary collaboration and a multimodality strategy are essential for optimizing patient outcomes. This review provides a surgical perspective on the management of GC across all stages, emphasizing the importance of a comprehensive approach.

### 1.1 Endoscopic resection

Endoscopic resection, including mucosal resection or submucosal dissection, has been adopted to treat clinical T1 patients with very low or no risk of lymph node metastasis [[4], [5], [6], [7], [8]]. The absolute indications for endoscopic resection include a differentiated mucosal (T1a) lesion without ulceration and tumor size >2 cm, a well-differentiated T1a lesion with ulceration and tumor size ≤3 cm, and an undifferentiated T1a lesion without ulceration and tumor size <2 cm. The expanded criteria cover a differentiated submucosal (T1b) lesion (<500 μm from the muscularis mucosae, SM1) without ulceration and tumor size ≤3 cm. Nakajima et al. reported that no lymph node metastasis was observed in tumors meeting the above-mentioned criteria [8]. Additionally, a nonrandomized trial of endoscopic submucosal dissection for undifferentiated mucosal GC revealed en bloc resection and curative resection rates of 99% and 71%, respectively, with a 5-year overall survival (OS) rate of 99.3% [6].

[Table 1] lists the advantages and disadvantages of surgery and endoscopic resection in treating early GC. Gastrectomy, including adequate lymphadenectomy for small T1 lesions, provides radical treatment, precise tumor staging, and excellent cure rates. However, surgery is a more invasive procedure, requiring partial or total stomach resection, which can impair a patient's quality of life and carries surgical complication risks. In contrast, endoscopic resection is less invasive, preserves the stomach, and has minimal impact on a patient's quality of life. Nonetheless, subsequent surgery may be necessary to achieve curative results if the endoscopic resected specimen reveals positive margins, lymphovascular invasion, or if the tumor exceeds the criteria for endoscopic resection, which may carry the risk of lymph node metastasis. Furthermore, endoscopic treatment may be an option for elderly patients with limited life expectancy or for high-surgical-risk patients with severe comorbidities.

**Table 1:** Advantages and disadvantages of surgery and endoscopic resection in treating early gastric cancer.

Approach	Advantage	Disadvantage
Surgery	Radicality precise staging	Sacrifice of stomach more invasive quality of life impairment

Endoscopic resection	Organ preservation less invasive minimal impact on quality of life	Need surgery for curability^a^
		Risk of lymph node metastasis

a Endoscopic resection specimen revealed positive margins, lymphovascular invasion or beyond criteria of endoscopic resection.

### 1.2 Extent of lymphadenectomy

**Fig. 1:**
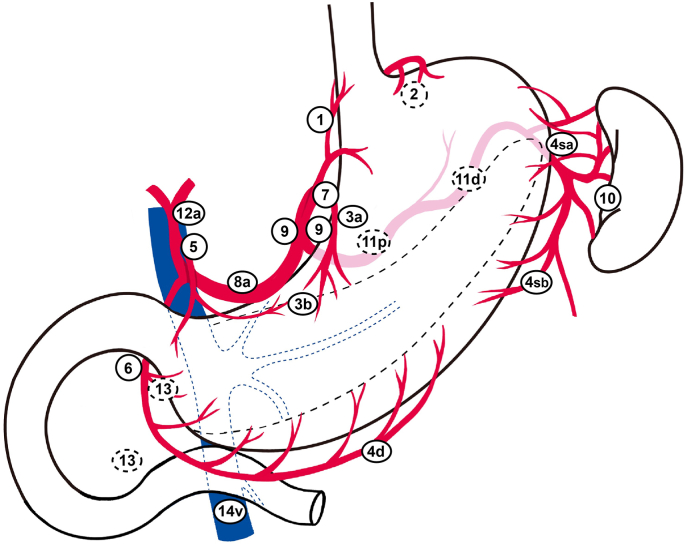
Lymph node location according to the Japanese gastric cancer classification (2011, 3rd edition)^37^.

Surgical treatment of GC typically involves gastrectomy and thorough lymph node dissection to achieve negative margins, so called R0 resection. [Fig. 1] and [Table 2] show the location of lymph node stations for GC according to the Japanese GC classification [37]. The lymphadenectomy is categorized as D1, D1+, or D2, depending on the level of lymph node removal. The depth of tumor invasion, nodal status, and tumor location determine the type of gastrectomy and the extent of lymphadenectomy [Table 2]. According to the Japanese gastric cancer treatment guidelines [4], D1 lymphadenectomy is recommended for clinical node-negative T1a tumors and differentiated type T1b lesions with a tumor size <1.5 cm. D1+ lymphadenectomy is suggested for clinical T1N0 tumors not meeting the above criteria, while D2 lymphadenectomy is indicated for tumors with clinical suspicion of nodal involvement or clinical ≥ T2 tumors.

**Table 2:** Location of lymph node stations and extent of lymphadenectomy according to the Japanese gastric cancer treatment guideline 2011 (3rd edition)^37^ and 2021 (6th edition)^4^.

Station No.	Lymph node location
1	right para-cardia
2	left para-cardia
3	Lesser curvature
4sa	Short gastric artery
4sb	Left gastroepiploic artery
4d	Right gastroepiploic artery
5	Supra-pylorus
6	Infra-pylorus
7	Left gastric artery
8a	Anterior to common hepatic artery
9	Celiac trunk
10	Splenic hilum
11p	Proximal to splenic artery
11d	Distal to splenic artery
12a	Proper hepatic artery
13	Posterior to pancreatic head
14v	Superior to mesenteric vein

Type of gastrectomy	Extent of lymphadenectomy	Lymph node station No.
Distal	D1	1, 3, 4d, 4sb, 6, 7
	D1+	D1 + 8a, 9
	D2	D1 + 8a, 9, 11p, 12a
Proximal	D1	1−3, 4sa, 4sb, 7
	D1+	D1 + 8a, 9, 11p
Total	D1	1–7
	D1+	D1 + 8a, 9, 11p
	D2	D1 + 8a, 9, 11p, 11d, 12a

In the past two decades, research has highlighted the importance of specific lymph node stations in advanced GC. Extended lymphadenectomies contribute to accurate disease staging; however, the impact on survival remains unclear [38]. The No. 10 splenic hilar lymph node has been excluded from the definition of D2 lymph node dissection in total gastrectomy [39]. Studies have demonstrated higher metastasis rates to the No. 13 node (posterior aspect of the pancreatic head) in cases of advanced GC with duodenal invasion compared to those without, prompting recommendations for additional removal of the No. 13 node, as it has therapeutic value equivalent to that of second-tier lymph node dissection [40,41]. Moreover, No. 13 nodes are now recognized as regional lymph nodes according to the 8th edition of the International Union Against Cancer TNM Classification and the 15th edition of the Japanese Classification of Gastric Carcinoma [42,43]. The survival impact of superior mesenteric venous lymph node dissection (No. 14v) in GC is still controversial. Several studies have found that the frequency of No. 14v node metastasis was associated with advanced tumor stage, tumor location (lower third), and poor histological differentiation [[44], [45], [46]]. Additionally, the No. 6 node status can predict No. 14v node metastasis [[44], [45], [46]]. Interestingly, Eom et al. demonstrated that the addition of No. 14v lymph node dissection did not improve OS in stage I/II GC, but this procedure prolonged OS in patients with clinical stage III/IV GC located in the middle or lower third of the stomach [47]. Furthermore, D2 plus No. 14v lymphadenectomy can increase OS and decrease lymph node recurrence in distal GC patients with stage IIIB/IIIC disease [45,46]. Dissection of No. 14v for distal stomach cancer with metastasis to the No. 6 lymph nodes (D2 + No. 14v) is recommended in the latest Japanese gastric cancer treatment guidelines [4].

The mediastinal lymph node metastasis rates for Siewert II gastroesophageal junction adenocarcinoma range from 18% to 40% [48,49]. Kurokawa et al. indicated that mediastinal lymph node metastasis correlated with the length of esophageal involvement, with the rate of No. 110 node metastasis being 0.9%, 6.4%, 10.8%, 20.8%, and 28.6%, respectively, based on the length of esophageal invasion (<1.0 cm, 1.1–2.0 cm, 2.1–3.0 cm, 3.1–4.0 cm, and >4.0 cm) [48]. Therefore, mediastinal lymph node dissection for Siewert II tumors with esophageal involvement <2.0 cm can be omitted. For esophageal involvement between 2.0 and 4.0 cm, extended lymphadenectomy (D2 + No. 110) via a transabdominal hiatal approach, rather than a transthoracic approach, is suggested due to comparable survival outcomes and lower pulmonary complications with the former approach [[49], [50], [51]]. A transthoracic approach may achieve complete removal of mediastinal lymph nodes and provide therapeutic benefits when esophageal invasion exceeds 4 cm [52].

While the optimal number of lymph nodes to retrieve remains undefined, a minimum of 15 nodes is recommended for accurate staging by the Union for International Cancer Control (UICC)/American Joint Committee on Cancer (AJCC) [53]. Our previous studies indicated that retrieving more than 25 lymph nodes yielded more favorable outcomes compared to retrieving fewer than 25 nodes in node-negative advanced GC [54]. Liu et al. demonstrated that retrieving more than 25 lymph nodes was associated with the most favorable OS in stage II/III or node-positive GC [55]. Investigators recommend that lymph node retrieval exceed 25 nodes in advanced GC to ensure accurate staging and better local control [56,57]. Furthermore, a systematic review by Seevaratnam et al. supported our findings, showing that examining more lymph nodes reduced stage migration and possibly improved long-term prognosis [38].

Additionally, Huang et al. recruited a cohort of patients with a higher proportion of locally advanced GC who underwent radical gastrectomy to evaluate the utility of the metastatic-to-examined lymph node ratio (LNR) in further stratifying N3b disease (number of metastatic nodes >15) [58]. Their results indicated that LNR was the most powerful prognostic factor for N3b disease in multivariate analysis, suggesting its potential role in survival stratification for this patient group. Moreover, LNR could mitigate the effects of inadequate lymphadenectomy and prevent incorrect tumor staging, which could otherwise lead to stage migration [59].

These findings suggest that the more lymph nodes harvested by the surgeon or examined by the pathologist, the greater the radicality of lymphadenectomy and the accuracy of nodal status. A related concern is that surgical morbidity or mortality may increase with higher lymph node retrieval. However, studies have shown that experienced or high-volume GC surgeons and centers had comparable surgical complication rates between groups retrieving 16–25 lymph nodes and those retrieving more than 25 nodes [55].

### 1.3 Impact splenectomy on survival

Radical gastrectomy, including D2 lymphadenectomy, is the established procedure for treating advanced GC in East Asia [4,60]. Splenectomy, which involves the removal of lymph nodes at the distal splenic artery (No. 11d) or splenic hilum (No. 10), provides definitive radicality. However, studies have shown that patients undergoing total gastrectomy (TG) plus splenectomy experience significantly higher surgical morbidity and mortality rates compared to those undergoing TG alone [12], which may adversely impact long-term survival [61,62]. In this context, Csendes et al. observed comparable 5-year survival rates between the TG and TG plus splenectomy groups [62]. Similarly, Yu et al. found no significant improvement in 5-year survival in the splenectomy group [12].

For advanced proximal GC, TG with spleen preservation may suffice, provided the tumor does not involve the greater curvature, as corroborated by a randomized controlled trial by Sano et al. [63] and supported by our previous research [64]. Furthermore, a meta-analysis of randomized controlled trials concluded that splenectomy should not be performed for proximal GC, as it increases operative morbidity without improving OS compared to spleen-preserving procedures [65]. [Table 3] summarizes key studies addressing the survival impact of splenectomy on GC patients Furthermore, the Japanese GC treatment guidelines 2018 (5th edition) similarly advise against No. 10 lymphadenectomy for advanced GC when the tumor does not infiltrate the greater curvature [39]. Therefore, routine or prophylactic splenectomy is not recommended unless the spleen is involved or significant hilar lymphadenopathy is observed.

**Table 3:** Key studies addressing the survival impact of splenectomy on gastric cancer patients.

Study	Randomized	Tumor location	Procedure	5-year survival rate
Yu^6^	yes	Proximal	TG vs. TG-S	48.8% vs. 54.8%; *p* = 0.503
Csendes^61^	yes	Proximal	TG vs. TG-S	36% vs. 46%; p > 0.5
Sano^62^	yes	Proximal	TG vs. TG-S	76.4% vs. 75.1%
		Non-greater curvature		HR, 0.88 (CI: 0.67−1.12)
Wang^63^	No	Non-distal	TG vs. TG-S	51% vs 47%; *p* = 0.181
Marano^64^	Yes meta-analysis	Proximal	TG vs. TG-S	OS: No difference; *p* = 0.277
				RR, 0.92 (CI:0.79−1.06)

Abbreviations: CI: confidence interval; HR: hazard ratio; LN: lymph node; OS: overall survival, RR: risk ratio; TG: total gastrectomy; TG-S: TG-splenectomy.

### 1.4 Minimally invasive surgery

Significant advancements in surgical devices and techniques have led to the widespread adoption of minimally invasive surgery (MIS) in GC treatment [13,14,66]. Since the pioneering report of laparoscopy-assisted distal gastrectomy by Kitano et al., in 1994 [67], the application of laparoscopic approaches for GC has continued to evolve and expand. One of the primary challenges of MIS is its steep learning curve. Studies have reported an estimated learning curve of 20–40 cases for laparoscopic distal gastrectomy and up to 100 cases for TG [68,69]. RG, first reported by Hashizume et al., in 2003 [70], has been rapidly adopted by laparoscopic surgeons due to its technical advantages, including a stable three-dimensional stereoscopic high-definition view with magnification, complete tremor filtering, fine articulated movements of robotic arms with seven degrees of freedom, a shorter learning curve, and improved surgeon dexterity [15,71,72].

Large-scale randomized trials have confirmed the non-inferiority of long-term oncological outcomes, safety, and feasibility of LG for both early and advanced GC compared to conventional open gastrectomy (OG) [[73], [74], [75]]. However, caution is warranted when interpreting these findings, as patients with cT4b cancer requiring multiorgan resection were typically excluded from these studies [76]. Studies have suggested that the significant advantages of MIS, including laparoscopic or robotic approaches over OG, include reduced postoperative morbidity, intraoperative blood loss, incisional pain, early ambulation, and shorter hospital stays when performed by experienced surgeons [73,77]. Moreover, the largest meta-analysis comparing the short- and long-term outcomes of LG and RG for GC indicated that RG appears feasible and safe, with comparable oncological outcomes to LG [13]. However, RG is associated with significantly higher costs and longer operative times [13,14]. The benefits of RG should be validated through randomized controlled trials, as retrospective studies are prone to a high risk of bias. Although MIS offers beneficial effects compared to conventional OG, the costs, the surgeon's skill and experience, as well as the capabilities of the healthcare facility, should be comprehensively considered when determining the appropriate surgical approach.

### 1.5 Surgical strategies for locally advanced GC

Patients with locally advanced resectable GC presenting with esophageal invasion, duodenal invasion, or involvement of adjacent organs such as the pancreas, transverse colon, spleen, or liver often require extensive surgical interventions. These may include thoracotomy to remove the involved lower esophagus, pancreaticoduodenectomy (PD), or resection of other involved organs to achieve negative margins. However, studies have shown that patients undergoing multiorgan resection experience significantly higher surgical complications compared to those undergoing less extensive surgeries [3,78,79]. A systematic review article with *meta*-anlaysis also suggests that patients undergoing extensive surgeries involving resection of adjacent organs tend to have poorer outcomes [3].

Furthermore, patients who undergo PD often face a prolonged postoperative recovery, leading to a decline in general performance status, which may delay the administration of adjuvant chemotherapy. Previous research, including our study, has demonstrated poor outcomes for patients with pancreatic invasion who undergo gastrectomy combined with pancreatic resection—whether PD or distal/subtotal pancreatectomy—compared to those undergoing less extensive surgeries [79,80]. Notably, local recurrence rates do not decrease after gastrectomy and PD compared to gastrectomy alone (non-PD), suggesting that systemic therapy plays a crucial role in managing these patients [81]. Additionally, patients undergoing combined pancreatic resection, particularly PD, have worse outcomes compared to those undergoing resection of other adjacent organs [79]. These observations highlight that pancreatic involvement in GC signifies not only anatomical invasion but also more aggressive tumor biology. Consequently, alternative treatment strategies, such as neoadjuvant chemotherapy followed by surgery, should be considered for these patients to improve their prognoses.

### 1.6 Neoadjuvant chemotherapy

Adjuvant chemotherapy following radical gastrectomy with D2 lymphadenectomy has traditionally been the standard treatment for pathological stage II or III GC in Asia [26]. However, patient survival remains suboptimal, particularly for those with stage III disease, and adjuvant chemotherapy is often deferred or not administered post-gastrectomy due to surgical complications and poor patient performance status. In this context, neoadjuvant chemotherapy presents an attractive option for advanced GC, offering advantages such as intensifying chemotherapy and initiating treatment when the patient is in a fitter condition. This approach aims to downstage the primary tumor and achieve higher R0 resection rates. Studies have demonstrated the benefits of neoadjuvant therapy in resectable GC or gastroesophageal junction adenocarcinoma [[16], [17], [18], [19]]. [Table 4] summarizes the inclusion criteria, neoadjuvant regimens and outcomes in the pivotal trials. An open-label, phase 3 randomized trial involving cT4aN + M0/cT4bNanyM0 GC or gastroesophageal junction adenocarcinoma patients showed significantly longer 3-year disease-free survival (DFS) in the neoadjuvant chemotherapy [oxaliplatin and S-1 (SOX)] group compared to the adjuvant chemotherapy [oxaliplatin and capecitabine (XELOX) or SOX] group [59.4% vs. 51.1%; hazard ratio (HR), 0.77; 95% confidence interval (CI), 0.61–0.97; *p* = 0.028] [17]. Tian et al. demonstrated that neoadjuvant chemotherapy with docetaxel, oxaliplatin, and capecitabine (DOX) significantly improved 3-year OS (56.9% vs. 44.6% vs. 34.7%) and DFS (45.2% vs. 40.2% vs. 28.4%) rates compared to neoadjuvant XELOX regimens or upfront surgery for potentially resectable cT3-4NanyM0 GC [18]. Furthermore, patients treated with DOX showed significantly longer 3-year OS than those treated with XELOX (*p* = 0.037). Similarly, Kang et al. found that the addition of neoadjuvant chemotherapy with docetaxel, oxaliplatin, and S-1 followed by surgery and S-1 prolonged OS (adjusted HR, 0.72; 95% CI, 0.54–0.97; *p* = 0.028) and PFS (adjusted HR, 0.71; 95% CI, 0.53–0.94; *p* = 0.019) compared to upfront surgery and adjuvant S-1 in Asian patients with locally advanced GC [16]. In addition, a review article with meta-analysis has supported the efficacy of neoadjuvant chemotherapy in improving patient survival and surgical outcomes compared to adjuvant therapy [19]. Neoadjuvant therapy has been shown to increase the pathological complete response rate, achieve higher rates of R0 resection, and downstage disease severity, as evidenced by pathological responses in tumors and involved lymph nodes [19]. Taken together, neoadjuvant therapy represents a promising treatment option for patients with locally advanced GC or gastroesophageal junction adenocarcinoma, offering the potential to downstage tumors and improve patient prognoses.

**Table 4:** Pivotal trials of neoadjuvant chemotherapy in resectable gastric cancer.

Trial	Inclusion criteria	Regimen	Outcomes
Kang^10^	cT2-3N + M0 or T4NanyM0	DOX vs. upfront surgery	OS (adjusted HR, 0.72; *p* = 0.028); PFS (adjusted HR, 0.71; *p* = 0.019)
Zhang^11^	cT4aN + M0 or cT4bNanyM0	SOX vs. adjuvant XELOX or SOX	3-year DFS: 59.4% vs. 51.1%; HR, 0.77; *p* = 0.028
Tian^12^	cT3-4NanyM0	DOX vs. XELOX vs. upfront surgery	3-year OS: 56.9% vs. 44.6% vs. 34.7%; *p* = 0.0089

Abbreviations: DFS: disease free survival; DOX: docetaxel, oxaliplatin and S-1; HR: hazard ratio; OS: overall survival; PFS: progression free survival; SOX: oxaliplatin and S-1; XELOX: oxaliplatin and capecitabine.

### 1.7 Palliative surgery

Palliative non-curative gastrectomy is offered to patients with locally advanced GC or mGC who are deemed incurable, in order to relieve tumor-related symptoms such as bleeding, perforation, or obstruction. In surgically fit patients with gastric outlet obstruction, laparoscopic or open gastric bypass with gastrojejunostomy is preferable over endoscopic stenting due to lower rates of recurrent symptoms [82]. Additionally, placement of a venting gastrostomy and/or feeding jejunostomy tube may also be considered for patients with relatively higher surgical risk. Bypass surgery or palliative resection has been shown to maintain a better quality of life and improve oral intake, with a favorable prognosis [25,83]. However, questions remain regarding patient selection for palliative gastrectomy [84]. Our data suggest that patients with favorable prognostic factors, such as age ≤58 years, better preoperative nutritional status, less nodal involvement, and favorable general performance status, may benefit from palliative gastrectomy [26]. Furthermore, patients treated with palliative resection and salvage chemotherapy had better outcomes than those who received other management strategies [25]. A retrospective study involving a large cohort of mGC patients also demonstrated that gastrectomy combined with chemotherapy resulted in more favorable survival rates compared to other treatment options [85].

However, a subgroup of patients undergoing resection of the primary tumor and metastatic organs, such as the liver or ovary, to achieve R0 surgery had favorable prognoses [86,87]. On the other hand, Tokunaga et al. found that patients with peritoneal metastasis (PM) did not benefit from palliative gastrectomy [88]. Moreover, the REGATTA trial indicated that mGC patients with a single non-curable factor (confined to the liver, peritoneum, or para-aortic lymph nodes) who underwent gastrectomy (restricted to D1 lymphadenectomy without resection of metastatic lesions) followed by salvage chemotherapy had comparable survival to those receiving chemotherapy alone [24]. The discrepancy in the results mentioned above might partly be explained by highly selected patient populations and nonuniform treatment strategies among studies. It is important to weigh the benefits and risks of palliative resection, as higher surgical mortality rates were observed compared to non-resection or radical surgery [25]. We recommend palliative surgery for patients who meet the following criteria: Eastern Cooperative Oncology Group (ECOG) performance status ≤2, younger age, tumor-associated symptoms (such as bleeding, obstruction, or perforation), a peritoneal cancer index (PCI) score ≤10, or the achievement of completeness of cytoreduction (CC0/1) [Table 5].

**Table 5:** Suggested criteria for patients undergoing palliative surgery or conversion surgery.

Surgery	Recommended criteria
Palliative	ECOG ≤2; young; symptoms (bleeding, obstruction, perforation); PCI score ≤10; CC0-1 achieved
Conversion	ECOG ≤2 and downstaging

Abbreviations: CC: completeness of cytoreduction; ECOG: Eastern Cooperative Oncology Group; PCI: peritoneal cancer index.

### 1.8 Conversion surgery

The mainstream treatment for mGC primarily involves systemic therapy, such as palliative chemotherapy, targeted therapy, and/or immunotherapy [26,27]. However, prolonged systemic treatment may lead to acquired chemo- or immuno-resistance, and cumulative therapy-associated side effects could impact the efficacy of chemotherapy or lead to treatment discontinuation in responsive patients [89]. In this context, conversion surgery emerges as a novel therapeutic approach within a multimodal treatment strategy for select mGC patients. Conversion surgery involves potentially curative resection following systemic therapy for initially unresectable or borderline resectable tumors, whether due to technical or oncological reasons [90]. Several studies have demonstrated the survival benefits of conversion surgery in carefully selected patients [[21], [22], [23],^90^]. A review by Du et al. suggested the feasibility and benefits of this approach in suitable candidates [91].

Additionally, our previous findings indicated that conversion surgery significantly prolonged median OS compared to upfront surgery plus palliative chemotherapy or upfront surgery alone (23.4 vs. 13.7 vs. 5.6 months). Furthermore, patients who experienced downstaging (pathological stage I–III) had better prognoses than those who did not (stage IV) (30.9 vs. 18.0 months) [20], consistent with reports by Yamaguchi et al. [90]. Recent studies, such as the Neo-REGATTA trial, have prospectively evaluated the outcomes of mGC patients with a single non-curable factor who did not progress after systemic therapy and underwent radical resection followed by continuous chemotherapy [92]. Their results indicated longer PFS and OS in the resection group compared to the non-resection group [92]. Therefore, careful patient selection for conversion surgery is crucial. Yoshida et al. recommended that resectable tumors without PM are the optimal candidates for conversion therapy [93]. Kim et al. found that R0 conversion surgery was achieved in 10 of 43 patients (23.3%) with PM and identified that patients with initial non-curable lymph node metastasis had the best outcomes, while PM was associated with a poor prognosis [94]. Other authors suggested that pathological tumor size and R0 resection were independent prognostic factors [95,96]. Shin et al. also identified factors such as body mass index at diagnosis, HER2 status, microsatellite instability status, and the use of targeted agents as independently associated with favorable survival outcomes [97]. While existing studies provide valuable insights, a randomized, well-designed trial is necessary to confirm the benefit of conversion surgery for mGC patients, as previous studies have recruited limited case numbers. We suggest performing conversion surgery for mGC patients with ECOG ≤2 and downstaging after systemic treatments [Table 5].

### 1.9 Prevention and treatment of peritoneal metastasis

PM represents a significant challenge in the management of advanced GC, with a high incidence rate after radical surgery, ranging from 29% to 54% [98,99]. PM rates at diagnosis are approximately 15%, with a median survival time of 2.8–6 months [98,100,101]. Various risk factors contribute to the development of peritoneal recurrence, including advanced pathological T-stage (pT ≥ 3), lymph node involvement, poorly cohesive type tumors, undifferentiated histology, diffuse type, signet ring cell carcinoma, presence of perineural or angiovascular invasion, younger age, and female gender [89,[102], [103], [104]]. Surgery itself, particularly lymphadenectomy during gastrectomy, can also lead to peritoneal tumor dissemination, especially in patients with advanced T and N status [105].

HIPEC involves heating the chemotherapy solution to a temperature of 41–43 °C and infusing it into the peritoneal cavity to enhance its therapeutic effect by increasing the penetration of chemotherapy into cancer cells and enhancing the immune response [106]. Prophylactic (adjuvant) HIPEC has shown promise in reducing peritoneal recurrence rates and improving survival outcomes in high-risk patients [35,107].

Since palliative intravenous chemotherapies are poorly effective against GC with PM due to the diffuse tumor burden, the plasma-peritoneal barrier, and poor vascular delivery to the peritoneal space, their clinical benefits are limited, and survival outcomes are poor [98]. Increasing evidence shows that for patients with limited PCI, HIPEC combined with CRS has demonstrated safety and feasibility, leading to improved oncological outcomes [32,33,108]. Notably, the completeness of cytoreduction (CC0 or CC1) is an important prognostic factor [39]. However, for GC patients with severe PM (PCI >10) or extensive involvement of the small bowel mesentery, achieving CC0 or CC1 with CRS is complex and often impossible [109]. PIPAC is a novel and feasible intraperitoneal drug delivery method that involves administering low-dose chemotherapeutic agents as a pressurized aerosol [110]. This technique offers several pharmacokinetic advantages, including low toxicity, high intraperitoneal concentration, low systemic concentration, homogeneous intraperitoneal distribution, and deeper tissue penetration [111]. PIPAC could offer a valuable alternative for patients with unresectable peritoneal disease. A systematic review showed that the estimated 12-month survival rate from the first PIPAC was 25% (95% CI 0.05–0.51), which is longer than that of patients treated with systemic chemotherapy, where survival does not exceed 10.7 months (95% CI 9.1–12.8) [112,113]. A multicenter cohort study of 586 patients with PM reported a median survival of 15.4 months from diagnosis and 20.1 months for patients receiving more than 3 cycles of PIPAC [110].

Strict patient selection for PIPAC is essential to avoid ineffective treatment and reduce severe surgical complications. Experts recommend avoiding PIPAC for patients with a life expectancy of less than 3 months, a recent history of intestinal occlusion, intolerance to oral feeding or need for parenteral nutrition, and poor performance status with ECOG >3 [112]. Eveno et al. are conducting a randomized, controlled, multicenter phase II trial (PIPAC EstoK 01) to evaluate PIPAC in addition to intravenous chemotherapy, and results are awaited [114]. Additionally, ongoing research is assessing the role of PIPAC as a neoadjuvant treatment for curative surgery, with some studies showing potential for conversion to secondary CRS and HIPEC [110].

## 2 Conclusion

**Fig. 2:**
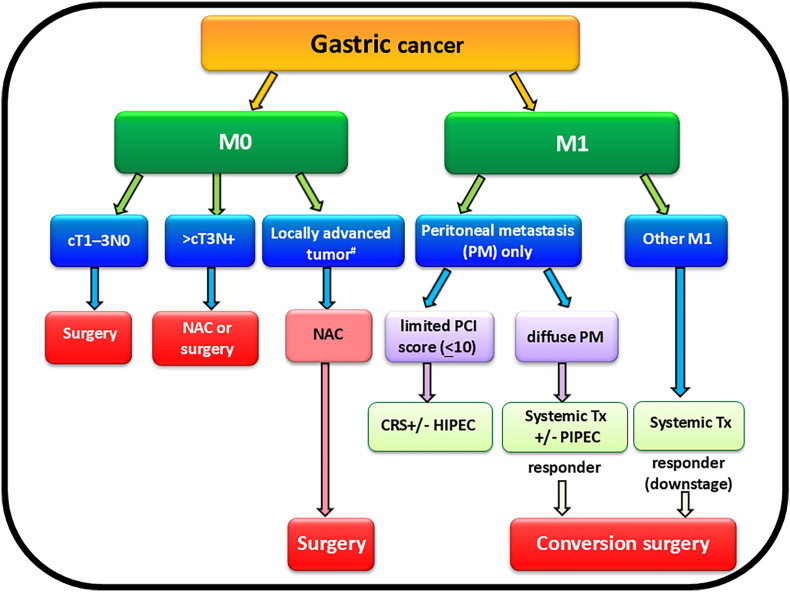
Algorithm of surgical treatment strategies for gastric cancer Abbreviations: #: invasion to the pancreas/duodenum/esophagocardia junction; CC: completeness of cytoreduction; CRS: cytoreductive surgery; HIPEC: hyperthermic; intraperitoneal chemotherapy; NAC: neoadjuvant chemotherapy; PCI: peritoneal cancer index; PIPEC: pressurized intraperitoneal aerosolized chemotherapy; PM: peritoneal metastasis; Tx: treatment.

We propose our surgical treatment strategies for GC shown in [Fig. 2]. The landscape of surgical management for GC is continuously evolving, with advancements in surgical techniques and systemic therapies. Early GC is typically treated with gastrectomy and D1 or D1+ lymphadenectomy (without clinical lymph node metastasis), while D2 lymphadenectomy is recommended for suspicious lymph node metastasis or advanced tumors. Endoscopic resection may be a viable option for early GC in patients with minimal risk of lymph node metastasis, particularly in elderly patients with high surgical risk or severe comorbidities. Spleen-sparing gastrectomy is emerging as a favorable option, offering reduced surgical complications and improved quality of life without compromising oncologic outcomes. MIS has become a safe and feasible alternative to conventional open gastrectomy, providing better short-term outcomes and comparable long-term prognosis.

Locally advanced GC poses challenges to achieving optimal survival outcomes, especially in cases of pancreatic invasion or lower esophageal involvement. Extensive surgeries like gastrectomy combined with pancreatectomy have not demonstrated significant improvements in survival. Neoadjuvant therapies, including chemotherapy or chemoradiation, have shown promise in downstaging the disease, increasing the likelihood of achieving R0 resection rates, and improving DFS and OS rates. For mGC patients who respond well to systemic therapies, conversion surgery offers a potential survival benefit.

Treatment of PM remains challenging, but prophylactic HIPEC has shown promise in reducing PM incidence in high-risk patients after radical surgery. HIPEC combined with complete CRS may prolong survival in carefully selected patients with low PCI scores, while PIPAC presents an alternative for patients with higher PCI scores. Surgeons must remain vigilant regarding ongoing investigations into novel surgical concepts and approaches, making informed decisions about patient selection and timing of interventions. Collaborative efforts within multidisciplinary teams are essential to optimize patient outcomes in the management of GC.

## Declaration of competing interest

The authors have no ﬁnancial or ethical conﬂicts of interest to declare.
