# Supplementary material for: A comprehensive overview of gastric cancer management from a surgical point of view
Source: Biomed J. 2024 Nov 18;48(4):100817. doi: 10.1016/j.bj.2024.100817 (PMC12320540; doi:10.1016/j.bj.2024.100817)
Supplement: Multimedia component 1 [file mmc1.docx]

**Supplement**

**Impact of surgery and surgical approach on the elderly**

With modern advanced medicine and improvements in medical care, human life expectancy has significantly increased. The treatment of elderly gastric cancer (GC) patients has become an increasingly important issue in aging society. Elderly individuals, especially those with severe comorbidities or reduced functional status, may be managed with a conservative attitude and less aggressive interventions. Currently, the most widely utilized treatment guidelines do not definitely include age as an independent factor to consider. Decision-making for treatment should contain the patient’s general performance status, overall health conditions, individual preferences, and quality of life concerns. Additionally, studies have suggested that “functional” age rather than “chronological age” should be the criterion for GC treatment [1].

A higher rate of comorbidities in elderly patients poses a higher risk of surgical morbidity and mortality [2–4]. A systemic review and meta-analysis of 26 studies recruiting 2030 GC patients between 1997 and 2017 concluded that patients aged 80 years and older had higher rates of surgical morbidity (risk ratios, 1.25, 1.09 to 1.43; p=0.001) and mortality (risk ratios, 2.51, 1.81 to 3.49; p=0.0001) compared to their younger counterpart, and significantly worse OS was observed in the older group [4]. In contrast, our previous results showed similar oncological outcomes between GC in the oldest old (>80 years old) and the younger (<80 years old) in the open gastrectomy era, which might be due to highly selected octogenarians with mild comorbidities undergoing surgery by experienced and dedicated surgeons in a high volume and academic facility [5]. Consistent with our findings, other studies also indicated no significant difference in surgical morbidity and mortality rates in terms of age (aged>80 years vs <80), especially without comorbidities supporting that the selection criteria are crucial to improving outcomes [6,7].

Minimally invasive surgery (MIS) has been demonstrated to offer better short-term postoperative outcomes than open surgery because of its minimal invasiveness [8–11], especially for the elderly, with evidence of showing fewer surgery-associated pulmonary and cardiac complications compared to the open approach [12,13]. In addition, a decrease in postoperative morbidity, probably related to less intraoperative blood loss, lower requirements for analgesics use and earlier ambulation, is important for better recovery in elderly patients. Nevertheless, studies have revealed that older age and comorbidity are associated with a higher incidence of systemic complications after laparoscopic gastrectomy (LG) [14]. Although robotic gastrectomy (RG) was introduced to overcome the limitations of LG, the longer operative time in RG than in LG raised concerns about potential hazards to elderly patients. In this regard, Okumura et al. [110] enrolled GC patients undergoing MIS in a high-volume center and reported outcomes of the elderly (>70 years old; RG, n=49 and LG, n=132) and younger (<70 years old; RG, n=321). Their results indicated that more comorbidities were noted in the elderly RG group compared to the younger RG group; however, surgical complications, pathological parameters, and disease-speciﬁc survival were comparable between the two RG groups. Compared to their LG counterparts, the elderly RG group had a longer operation time with comparable postoperative complications. There was no difference in overall survival and disease-free survival between the elderly groups. After multivariate analysis, age and surgical approach were not significant risk factors for overall and major complications. They concluded that RG seems to be a safe and feasible approach in elderly patients [15]. Thus, MIS is an ideal way to improve surgical outcomes for fit elderly patients under the care of experienced surgeons.
